# Psychiatric Disorders and their Association with Liver Transplant Outcome In Iran

**Published:** 2014-11-01

**Authors:** A. Sahraian, M. Sharifian, B. Geramizadeh, S. A. Malek-Hosseini

**Affiliations:** 1 Psychiatry Research Center, Hafez Hospital,; 2 Department of Neurology, Student Research Center,; 3 Transplant Research Center, Nemazi Hospital, Shiraz University of Medical Sciences, Shiraz, Iran

**DEAR EDITOR**

Liver transplantation is a lifesaving operation and the treatment of choice for patients with end-stage hepatic failure. Poor psychological status is recognized as a major contributor to morbidity, mortality, decreased quality of life, higher medical cost, and overutilization of health care services among transplant recipients [1-3]. 

In this study, conducted between 1999 and 2010, 260 liver transplant recipients who underwent the operation in Shiraz, the first and only center in Iran for liver transplantation, were studied. A psychiatrist evaluated the participants for any psychiatric disorders within a month before transplantation. In addition, pre-operative diagnosis, sex, age, marital status of recipients, type of operation, duration of hospitalization, rate of post-operative infections, and post-operative morbidity and mortality were recorded. The data was analyzed by SPSS^®^ for Windows^®^ ver 15.

The mean±SD age of 260 participants was 36.8±12.3 years. Of 260 participants, 151 (58%) were male. The pre-operative psychiatric assessment revealed that 53 (21%) patients had major depressive disorders; 6 (2%) had insomnia, 3 (1.5%) cognitive disorders, and 9 (3.5%) had other psychiatric disorders.

The prevalence of major depressive disorders was significantly (p<0.05) higher in patients with autoimmune hepatitis (n=21, 17%) than those with other causes of hepatic failure. The prevalence in women (n=35, 32%) was significantly (p<0.05) higher than that in men (n=18, 11%), an observation similar to that reported in other populations [4].

The mean hospitalization length was significantly (p<0.05) longer in patients who had pre-operative major depressive disorders than those without (25 *vs* 17 days).

Overall, 22 (8.4%) patients developed post-operative infections at any sites. The rate of infection in those with pre-operative major depressive disorders was significantly (p<0.05) higher than those without. The rate of graft rejection, post-operative malignancy, and mortality in 1-year post-operative in patients with and without pre-operative major depressive disorders did not significantly different.

Depression was the most important psychiatric disorder diagnosed in our patients before transplantation. Pre-operative depression may prolong hospitalization after transplantation and subsequent morbidities such as post-operative infections. Therefore, transplant patients with pre-operative psychiatric disorders should receive ongoing psychosocial counseling [5]. Suitable pre-medication, optimal psychosocial assessment, and treatment, especially for the women who carry a higher risk of depression are imperative [4].
